# High Adherence to Mediterranean Diet Is Not Associated with an Improved Sodium and Potassium Intake

**DOI:** 10.3390/nu13114151

**Published:** 2021-11-19

**Authors:** Giulia Viroli, Carla Gonçalves, Olívia Pinho, Tânia Silva-Santos, Patrícia Padrão, Pedro Moreira

**Affiliations:** 1 Facoltà di Alimentazione e Nutrizione Umana, Università degli Studi di Milano, 20133 Milano, Italy; giulia.viroli@studenti.unimi.it; 2 Faculdade de Ciências da Nutrição e Alimentação, Universidade do Porto, 4150-180 Porto, Portugal; oliviapinho@fcna.up.pt (O.P.); taniiasilvasantos@gmail.com (T.S.-S.); patriciapadrao@fcna.up.pt (P.P.); pedromoreira@fcna.up.pt (P.M.); 3 CITAB—Centre for the Research and Technology of Agro-Environmental and Biological Sciences, Universidade de Trás-os-Montes e Alto Douro, 5000-801 Vila Real, Portugal; 4 CIAFEL—Centro de Investigação em Atividade Física, Saúde e Lazer, Universidade do Porto, 4200-450 Porto, Portugal; 5 Laboratório para a Investigação Integrativa e Translacional em Saúde Populacional (ITR), 4050-600 Porto, Portugal; 6 LAQV-REQUIMTE—Laboratory of Bromatology and Hydrology, Faculty of Pharmacy, University of Porto, 5000-801 Porto, Portugal; 7 UP EPIUnit—Institute of Public Health, Universidade do Porto, 4200-450 Porto, Portugal

**Keywords:** salt, sodium, potassium, Mediterranean diet, 24-h urinary excretion

## Abstract

Prevention and control of hypertension and cerebro-cardiovascular diseases are associated with adequate sodium and potassium intake and adherence to a Mediterranean dietary pattern. The aim of this study was to assess the association between adherence to a Mediterranean diet (MD) and the excretion of sodium and potassium as surrogate measures of intake. This is a cross-sectional analysis as part of a larger study (the iMC SALT randomized controlled trial) among workers of a public university. A food frequency questionnaire was used to assess the adherence to MD, using the alternative Mediterranean diet (aMED) score; sodium and potassium excretions were estimated by 24-h urine collections. Sociodemographic and other lifestyle characteristics were also obtained. The associations between the adherence to MD and Na and K excretion were calculated by logistic regression, adjusting for confounding variables. From the 109 selected participants, seven were excluded considering urine screening and completeness criteria, leaving a final sample of 102 subjects (48% male, average age 47 years). Mean sodium and potassium excretion were 3216 mg/day and 2646 mg/day, respectively. Sodium and potassium excretion were significantly higher in men, but no differences were found according to different levels of MD adherence. In logistic regression analysis, sodium, potassium, and sodium-to-potassium ratio urinary excretion tertiles were not associated with MD adherence (low/moderate versus high), even after adjustment for confounding variables. A high adherence to MD was thus not associated with a different level of sodium and potassium intake.

## 1. Introduction

Cardiovascular disease (CVD) is the leading cause of disease in the world [1,2] causing an estimated 31% of worldwide deaths [3] and hypertension is one of the known major risk factors for CVD, in addition to lifestyle, physical inactivity, genetic and dietary factors [4]. In particular excessive dietary intake of sodium [5,6] is a leading determinant of high blood pressure and cardiovascular risk [7,8,9]. Several behavioural interventions have been prooosed to reduce sodium intake [10], but nevertheless most adult populations have an estimated mean sodium intake which exceeds 2.30 g/day (100 mmoL/day) [11], above the WHO recommendation <2 g/day (or <85 mmoL/day) [12]. In contrast, increased potassium intake has been shown to reduce systolic blood pressure and stroke events [13,14,15] and the WHO recommends a potassium intake of at least 3.51 g/day (90 mmoL/day) for adults [16]. The sodium-to-potassium (Na/K) ratio has also been considered a good predictor of hypertension and cardiovascular disease [17,18,19,20,21].

Indeed, diets high in sodium (>4 g/day) and low in potassium (<2 g/day) have been associated with increased BP [21]. The Mediterranean diet is an expression of a lifestyle described by Keys is the 1960s [22] which has been related to a wide range of different healthy outcomes, including lower BP and a decreased risk for cardiovascular events [23,24,25]. This dietary pattern is characterized by the use of olive oil as the main source of fat, a high intake of plant-based foods, including fruits, vegetables, whole grains and cereals and legumes, low to moderate red wine consumption, low consumption of meat and meat products and increased consumption of fish and moderate consumption of milk and dairy products.

Although it is expected that the intake of potassium and sodium may be adequate in the Mediterranean dietary pattern, several studies have investigated the relationship between the adherence to the Mediterranean diet and the intake of sodium, potassium and their ratio, finding inconsistent results. Furthermore, some of these studies estimated the intake of sodium and potassium from food frequency questionnaires (FFQ), a method that can hardly estimate accurately sodium intake, especially the amount added during the preparation of the meal or at the table [26], being recognized that 24-h urine collection is considered the gold standard to measure of sodium intake. Nevertheless, this method is also demanding, requiring great collaboration by the participants, and may be limited by under-collection and the lack of a conclusive methodology to accurately identify incomplete samples [27,28,29].

Kanauchi et al. [30] reported in Japan that high adherence to a Mediterranean dietary pattern was associated with a higher intake of salt and potassium estimated with FFQ. In another study, La Verde [31] reported that higher adherence to MD was not inversely associated with hypertension when corrected for sodium and potassium intake, suggesting that lower sodium intake could be a mediating factor of the MD effects to hypertension. Using 24-h urine collection to study this relationship, Parvin Mirmiran et al. [32] reported no significant relationship between adherence to a MD and sodium and potassium intake and their ratio, as did Vasara [33].

The aim of this study was to quantify sodium and potassium excretion (and their ratio) by 24-h urine collection and assess its association with the adherence to a Mediterranean dietary pattern in adults.

## 2. Materials and Methods

The subjects of this study are the participants involved in the iMC SALT randomized control study conducted in Porto (Portugal) from June 2019 to January 2021 [34]. The aim of this intervention is to reduce salt consumption among consumers using an innovative prototype device (Salt Control H—INPI, N° 20191000033265) that monitors and controls the amount of salt used when preparing and cooking food. A detailed description of the project has been reported elsewhere [34]. In brief, participants from the University of Porto were recruited by a physician during routine health appointments and, if eligible, signed the informed consent form and were randomly assigned to the intervention group (IG) or to the control group (CG). The intervention lasted 8 weeks, during which the participants use the Salt Control H equipment to dose salt when preparing and cooking food at home. During this study, a total of four visits, excluded the screening visit (visit 0), were conducted to monitor different outcomes. The primary outcome was the 24-h urinary sodium excretion, which has been measured during the four visits. Blood pressure and anthropometric data were also collected. Ethical approval was provided by the Ethics Committee of the Centro Hospitalar Universitário São João and good clinical practices regarding clinical trials with intervention were followed [35,36].

For the current study the data obtained at the baseline from the questionnaires (socio-economic; demographic; medical [37]; physical characteristic [38]); 24-h urine analysis and the dietary assessment carried out by FFQ has been used.

### 2.1. Study Participants

Participants were recruited from among the staff of University of Porto (both white-collar and blue-collar workers) by a doctor during routine occupational health appointments. The inclusion criteria of the participants selected were being an adult (>18 years), frequently eating home-cooked meals (more than four days a week and at least three Sundays per month), one annual occupational health appointment at the hospital and reported motivation to control dietary salt consumption. The exclusion criteria were being pregnant, with hypotension, kidney disease, active infection that impacts renal function, urinary incontinence, acute coronary syndrome, severe liver disease or heart failure, not using salt for cooking, and being a staff member of the faculty conducting the study. All participants signed an informed consent statement and received a document containing information about the study.

### 2.2. Urinary Sodium and Potassium

A 24-h urine sample was collected by each participant. Collections were performed using sterile containers provided to the participants during the visits and containers were coded to conceal their provenance. The 24-h urine collection procedure was explained to each participant and a pamphlet was given with indications about the procedure. On the day of collection, the participants discard the first urine and thereafter included all urine for the day, and the first urine on the following day. On receipt of the containers on the day immediately following the 24-h urine collection, the researcher sent the containers to a certified laboratory. The 24-h urine collection has been performed on any day of the week (preferably on Sunday, but not on Friday and Saturday due to laboratory availability).

The parameters analyzed were sodium (by indirect potentiometry), potassium (by indirect potentiometry) and creatinine (by photometry). The validity of the 24-h urine samples was assessed considering the ratio of urinary creatinine (mg/day) to body weight (kg) and samples were excluded for female when the creatinine (mg/d/kg) was <10.8 and >25.2 while for male when creatinine (mg/d/kg) was <14.4 and >33.6 [39].

In order to convert urinary output to dietary intake, the urinary intake of sodium (mg/day) was calculated considering that urine output reflect 90–92% of the intake (calculate with the proportion X intake = (excretion × 100)/91), while potassium intake was estimated considering that 77% of the potassium ingested is excreted in the urine [40]. The conversion of mg to mmol has been carried out dividing sodium (mg) and potassium (mg) respectively for 23 or 39 (mmol = mg/atomic weight). The conversion from dietary sodium (Na) intake to salt (NaCl) intake was made by multiplying the sodium value by 2.542 (NaCl (g) = Na (g) × 2.542). The prevalence of inadequacy of sodium and potassium intake was also calculated considering WHO recommendations [12,16].

### 2.3. Dietary Assessment and Adherence to the Mediterranean Diet

Dietary intake was assessed by a semiquantitative Food Frequency Questionnaire (FFQ) validated among Portuguese adults [41] and adapted to include a variety of typical Portuguese food items. The FFQ administered covers the previous 12 months before inclusion in the study. It includes 82 food items or beverage categories and nine possible options for the frequency of food consumption ranging from never/less than one a month to six or more time per day (never or less than 1 time per month; 1 to 3 times a month; once a week; 2–4 times a week; 5–6 times a week; once a day; 2–3 times per day; 4–5 times per day; and 6 or more times per day). To convert food intakes in grams we used the software Food Processor Plus (ESHA Research, Inc., Salem, OR, USA) adapted to the Portuguese population by using the values from Portuguese Tables of Food Composition for typical Portuguese foods [42]. The nutritional content of local food was taken from standard nutrient tables, whereas the content of commercial food (e.g., pizza and ready-to-eat-food) was derived from labelled ingredients and nutrients. For soup, 26% of this food has been considered for the calculation of the vegetable score considering that the recipe for 350 g of “Juliana soup” requires 90 g of vegetables (excluding potatoes).

The degree of adherence to the traditional Mediterranean diet was assessed through the alternative Mediterranean Diet (aMED) Score [43], a score developed on the basis of Mediterranean-Diet Scale by Trichopoulou [44]. aMED score was calculated for each participant from the estimation of food consumption measured with FFQ. The items included in this score were nine: vegetables, fruits, legumes, nuts, whole grains, fish, red meat and processed meat, ethanol and the ratio of monounsaturated fatty acids to saturated fatty acids (MUFA/SFA). For vegetables, legumes, fruits, nuts, whole grains, and fish, consider as beneficial components, were assigned a value of 1 when consumption of subjects was above the sex-specific median, while when consumption was below the median were assigned a value of 0. For red meat and processed meats, consider as detrimental components, was assigned a value of 1 when the consumption of subjects was below the sex-specific median or above the median were assigned a value of 0. Alcohol was the only component in which score has been assigned according to cut off, a value of 1 was assigned to men whose consumption was 5–25 g/day and women who consumed 5–15 g/day. Finally, MUFA/SFA was used to assess the quality of fat intake, giving a value of 0 to participants whose MUFA:SFA ratio was below the sex-specific median and a value of 1 to participants whose ratio was at or above the median. So, total score of a participant ranged from 0 (minimal adherence to the traditional Mediterranean diet) to 9 (maximum adherence). A low adherence to Mediterranean diet has been considered when the score was between 0–3, moderate 4–5, or high 6–9.

### 2.4. Other Measurements

Height (model 213 portable stadiometer, SECA, Hamburg, Germany) and waist and hip perimeter (SECA 201 tape) were measured according to international standard procedures [45] with participants wearing light clothing and without shoes. Body Mass Index (BMI) was calculated by dividing the weight (kg) by height squared (m).

Blood pressure was measured using a portable sphygmomanometer (Edan M3A, Edan Instruments, 15 Jinhui Road, Pingshan District, Shenzhen, 518122 P.R. China) with the participants seated and was taken after a 5 min rest. The measurements were done in both arms to determine possible arm-related measurement differences. Using the arm with the highest blood pressure value as a reference, the blood pressure was assessed twice. If the difference of systolic pressure between the first two evaluations was higher than 10 mm Hg, one more evaluation was done. The hypertension diagnostics was performed by the doctor responsible for the recruitment based on patient’s clinical record.

The level of physical activity has been characterised using the International Physical Activity Questionnaire-Short Form, validated and adapted to the Portuguese population [38]. Physical activities were divided into moderate, vigorous and walking in according to the type, energy expenditure require (MET), and the amount of time spent (min). For each participant has been calculated the total MET × min × week and the level of physical activity was considered “high” when the total MET × min × week was >3000; “moderate” >600 and the rest was defined as “low” [46].

The sociodemographic questionnaire was based on the WHO STEPS questionnaire [37] a standardized method to collect sociodemographic characteristic and to investigate the key behaviour risk factor (such as tobacco and alcohol use) for CVD. Participants were considered drinkers when the alcohol intake was above 25 g/day for males and 15 g/day for females.

### 2.5. Statistical Analysis

Baseline characteristics were presented as mean plus standard deviation for continuous variables; or frequency (%) for categorical variables. Differences between male and female were assessed using independent sample *t*-tests for continuous variables or χ^2^-tests for categorical variables.

Mann-Whitney test was used to compare food groups intake between high/low-moderate adherence to MD and between sex.

Separate binary logistic regression models were fitted (odds ratio (OR) with respective 95% confidence intervals (CI)) for male and female to examine the magnitude of the association between high Mediterranean Diet adherence (dependent variable, dichotomous variable low/moderate vs high adherence to MD), and sodium, potassium, and sodium-to-potassium ratio urinary excretion (independent variables categorized in tertiles), adjusting for age, BMI (Model 1), plus energy intake (Model 2), plus educational level, hypertension status and physical activity level (Model 3). For all comparisons, the significance level was set at 5%. All statistical analyses were conducted using SPSS Statistics for Windows, Version 26.0 (IBM Corp, Armonk, NY, USA).

## 3. Results

### 3.1. Characteristic of Participants

After the initial screening 109 participants provided the FFQ at the baseline; seven female participants were excluded because they did not meet the creatinine excretion criterion, so finally, 102 participants were analysed.

The final sample was 48% male. Mean age was 47 years old (range from 24 to 69) without significant differences between sexes. BMI was significantly different between male and female (27.2 ± 3.4 vs 24.5 ± 3.7 kg/m^2^, *p* < 0.001). Mean (±SD) systolic blood pressure was 126.6 ± 18.6 mm Hg, significantly higher in males (*p* < 0.001); also, diastolic blood pressure was significantly higher in males than females (*p* = 0.005) with a mean of 80.3 ± 14.5 mm Hg; hypertension was reported in 24.5% of men and 11.3% of women (Table 1).

### 3.2. Urinary Excretion, Salt Intake and Dietary Intake

Data regarding the general characteristics of participants is presented in Table 1. Nutritional and dietary intake considering the food groups for MD adherence score are presented in Table 2. In both sexes, the group of high adherence to MD had a statistically significant (*p* = 0.002) higher energy intake than the low to moderate category. Every food group median sex-specific intake was higher in participants with a higher adherence to the MD, except for red meat and alcohol intake. The frequency of low, moderate, and high adherence to MD was, respectively, 19, 15, 15 in males, and 20, 21, 12 in females.

The mean sodium urinary excretion was 3216 ± 1307 mg/day (8.98 g/d of salt), and potassium was 2646 ± 755 mg/day. No statistically significant differences were found when comparing mean sodium excretion in the low-moderate versus high adherence to MD category in both sexes (respectively, 3868 ± 1390 vs. 3807 ± 1179 mg/day, in men, and 2591 ± 966 vs. 2768 ± 1116 mg/day, in women).

In relation to potassium urinary mean excretion, like for sodium, no statistically significant differences were found when comparing low-moderate adherence versus high adherence to MD (respectively, 2930 ± 849 and 2938 ± 681 for males, and 2373 ± 660 and 2412 ± 443 mg/day for females). No significant differences were found for the urinary sodium-to-potassium ratio according to adherence levels to MD (Table 2).

As a result, the average salt intake was 8.98 ± 3.65 g/day without significant difference between categories of adherence to MD, but males had a significantly higher intake of salt than females (10.75 vs. 7.35 g/day). No associations were found for the distribution of salt intake according to WHO guidelines and MD adherence, in both sexes; 92% of the males with a low-moderate adherence to MD diet did not follow the WHO guidelines (<5 g of salt) and this percentage reached 100% in the males with high adherence, and for females the percentages were lower, respectively 65.9% and 58.3%.

Moreover, around 40% of males did not reach the WHO cut-off for adequate potassium intake (>3510 mg/day) regardless of their MD diet adherence, and for females the percentages were 68.3% and 75% in low-moderate and high adherence groups, respectively.

Considering the tertiles of excretion for the abovementioned variables (Table 3 and Table 4) in logistic regression models, no significant associations were found for sodium and potassium intake and MD adherence.

## 4. Discussion

This is one of the few studies [33] to investigate the relationship between sodium, potassium and ratio sodium-to-potassium excretion and intake, estimated from 24-h urine collection and Mediterranean diet adherence level in Portuguese adults. The intake of sodium and potassium across different levels of adherence to MD seems to be similar, considering that no significant differences have been found in different levels of MD adherence.

This indicated that Mediterranean diet, a well-known dietary pattern associated with several healthy outcomes, is not related to the amount of ingested salt, an important risk factor involved in the development of cardio and cerebrovascular diseases [7,8,9].

This finding is in accordance with results of the SING study [33] conducted in Greece where no association was found between the intake of sodium, potassium and their ratio (estimated with 24-h urine excretion) and the adherence to Mediterranean diet. Although we could expect that the contribution of potassium could be higher in a pattern of greater adherence to the Mediterranean diet, considering its plant-based component, no significant differences were found, which could be possibly due to a different contribution of some foods rich in potassium intake that were not captured in the scores. However, in another study [30] conducted in Japan, an association of a higher intake of favourable nutrients with the adherence to MD adapted for the Japanese population, occurred, with the exception of sodium intake. The estimation of dietary intake has been made with FFQ and this method could also have underestimated the amount of sodium ingested due to the inaccuracy and bias assessing salt added at the table and during cooking. Fujiwara [47] found in a Japanese population that the dietary pattern of “fish and vegetables” was correlated with healthy outcomes and, at the same time, to the amount of sodium ingested. The author suggested that the mains sources of sodium in this healthy dietary pattern were salted fish and sea products, pickled vegetables, miso soup and salt-containing seasonings. La Verde [31] found that the inverse relationship between adherence to MD and hypertension was no more significant after the adjustment for sodium intake. Instead, Mirmiran [32] found that Mediterranean score was inversely associated with urinary sodium-to-potassium ratio.

One factor that could influence the amount of sodium intake is the quantity of food ingested: the higher is the amount of food ingested, the higher is the sodium intake. In this study, the energy intake of the group with a high adherence to MD was significantly higher than the group with low to moderate adherence. However, in the logistic regression model after adjusting for energy intake, the relationship between the urinary excretion of sodium, potassium and their ratio was not significantly associated with the adherence to MD, even after adjustment for BMI. This analysis was also important considering the significantly higher urinary excretion of sodium, potassium, and their ratio in male than female, which persisted after adjusting for energy intake and BMI (data not shown). In the IAN-AF Portuguese survey, men had a higher intake of sodium too [48]. It would be interesting in future research to understand the differences in eating practices that could explain these sex differences in urinary excretion.

Mediterranean diet can be defined as a lifestyle where the eating habits are characterized by a high intake of vegetables, fruits and legumes, which are food groups naturally containing low amounts of sodium and high amounts of potassium. This combination could be the marker of a healthy dietary pattern. It has been associated with lower blood pressure and a lower risk of death and CV events [20,49] as well as Mediterranean diet [25].

However, when the adequacy of Mediterranean diet for sodium and potassium intake is evaluated, the type of score used has to be considered. The most used score, developed by Trichopoulou [44], does not take into consideration the amount of foods eaten according the types of food processing, including those with notorious amounts of salt. In addition, the amount of salt added at the table and during the preparation of the meal is not considered either. Both factors have an influence on sodium intake and could bring the level of salt intake above the recommended level, and similarly across different levels of adherence to MD, including for those subjects with a high score in MD adhesion. In this study 100% of the males and 58% of the females with a high adherence to MD were above the WHO recommendation for sodium; while male and female participants whose intake of potassium was above 3510 mg/day were respectively 41% and 70%. The mean sodium-to-potassium ratio was above the recommendation of 1 (mmol/mmol) for both sexes at every level of adherence of MD. Around the world salt intake came from different sources, depending on the geographical area. In the Western countries the main sources are processed foods, while in Asia is discretionary salt [11,50]. In the Portuguese population the main source of sodium is the salt added at the table (29.2%), followed by bread and toasts (18.0%), soup (8.2%) and salami and processed meat (7.0%) [48]. In this sample the contribution of soups to the vegetable intake was 33% and this is a type of food included in the score as a beneficial component.

The eating habits and PA of the participants in this study have to be contextualized in the period of COVID-19 pandemic. The restrictions adopted by the goverment to contain the spread of the virus led to the transition to online work for several workers, including academic stuff, and strictly limited social life. Two national surveys [51,52] analysed the impact of restriction on dietary habits and level of PA in the Portuguese population during the hard confinement finding that most participants reported changes in eating habits, whereby the number of meals cooked at home increased while delivery and ready to eat meal consumption decreased. In general, the quality of the diet improved (18.2% and 58%). Silva et al. reported that regarding the foods category, the amounts of sweet snacks (30.9%), fruits (29.7%), and vegetables (21.0%) increased; woman reported the highest increase in vegetables and sweet snacks consumption, while men reported the highest increase in fruit consumption. Soft drinks (32.8%), savoury snacks (30.9%), and alcoholic beverages (28.2%) were the food categories with the highest proportion of reported decrease in consumption. PA level reported in the same study increased during social confinement in comparison to the international surveys IAN-AF (2017), so because of COVID-19 confinement and the change of behaviour that followed, the participants recruited during 2020 (n = 38) could have reported dietary habits and PA levels different than usual.

One other issue to be taken in account when the role of sodium and potassium in the diet are evaluated is the method used for their estimation. The 24-h urine collection is the only method that can measure without reported bias the amounts of salt and potassium ingested and is considered the gold standard method by several authors to estimate sodium intake [52,53]. Nevertheless, although the standard method for assessing sodium and potassium intake is 24-h urine collection, which is based on the recognition that 24-h urinary sodium and potassium excretion may reflect at least 90% of sodium [54] and 70% of the ingested potassium [54], this approach also presents the limitation of the 24-h urinary excretion, which can differ significantly from the 24-h measured intake [55] and may present inaccuracies and collection errors [56].

It is important to underline that the benefits of the Mediterranean diet extend beyond the content of sodium and potassium. On the opposite side for example NO and antioxidant compounds have a beneficial effect on vascular function lowering the risk of CV [57]. MD is a sustainable and resilient dietary pattern [58] that respect environment, biodiversity, culture and skills of the population. Health and these factors are essential to develop a sustainable long-term diet for people and countries.

This finding highlights how the intake of salt is still a problem, and the intake of potassium could be below the recommendation. Further investigation on the amount of sodium and potassium ingested following a Mediterranean diet are needed to better understand the relationship.

The limitations of the study can be summarized in three points. First, the number of participants could be considered too low compared with the number of subjects in other similar studies [33]. Second, the results are not generalizable to the overall population because the sample was composed by adults with a high level of education, a small number of smokers and lower intake of salt than the average of the Portuguese population (the average salt consumption in Portuguese population is 10.7 g salt/day [59]). In addition, most of the participants came from an urban area.

Third, the nutritional assessment with 24-h urine collection is the best method for collective surveillance, but an agreement about the criteria to define a urine sample eligible has not been reached around the world. In fact, creatinine excretion is used to assess completeness of urine collection because it is considered to be less variable in urine than sodium, but it can also vary from day to day in relation to muscle mass, medicines being taken and eating habits, such as the consumption of meat [60].

## 5. Conclusions

The estimation of sodium and potassium excretion by 24-h urine collections showed that a high adherence to MD was not associated with a lower excretion of sodium or a high excretion of potassium, or their ratio, suggesting that this undoubtable healthy traditional dietary pattern was not associated with the recommended salt intake levels.

## Figures and Tables

**Table 1:** General characteristics of participants according to sex and adherence to a Mediterranean diet.

				Adherence to Mediterranean Diet					
				Male			Female		
General characteristic	Male *n* = 49	Female *n* = 53	*p*	Low-moderate *n* = 34	High *n* = 15	*p*	Low-moderate *n* = 41	High *n* = 12	*p*
Age ^a,b^	49 (31–69)	46 (24–64)	0.06	49 (31–69)	50 (31–62)	0.915	45 (24–64)	47 (34–54)	0.458
Educational level ^c,e^			0.227			1.0			0.679
Non university graduates	8.2%	13.2%		8.8%	6.7%		14.6%	8.3%	
University graduates	91.8%	86.8%		91.2%	93.3%		85.4%	91.7%	
Marital status ^c,e^			0.227			1.0			0.301
Not married	26.5%	37.7%		26.5%	26.7%		41.5%	25%	
Married	73.5%	62.3%		73.5%	73.3%		58.5%	75%	
Physical activity ^c,e^			0.866			0.157			0.233
Low	32.7%	37.7%		41.2%	13.3%		31.7%	58.3%	
Moderate	49%	45.3%		41.2%	66.7%		48.8%	33.3%	
Active	18.4%	17%		17.6%	20%		19.5%	8.3%	
Smoking status ^c,e^			0.425			0.159			1.0
Current smoker	12.2%	7.5%		17.6%	0%		7.3%	8.3%	
Drinking status			<0.001			0.298			0.87
Current drinker	91.8%	62.3%		88.2%	100%		56.1%	83.3%	
BMI ^d,a^	27.2 ± 3.4	24.5 ± 3.7	<0.001 ^b^	27.0 ± 3.6	27.8 ± 3.0	0.419	24.2 ± 3.5	25.4 ± 4.1	0.339
Systolic blood pressure (mmHg) ^d,b^	133.6 ± 19.7	120.2 ± 15.1	<0.01 ^b^	135.2 ± 20.5	129.9 ± 17.6	0.393	119.6 ± 15.7	122.1 ± 13.5	0.618
Diastolic blood pressure (mmHg) ^d,b^	84.5 ± 16.1	76.5 ± 11.7	0.005	85.1 ± 17.4	82.9 ± 13.2	0.664	76.2 ± 12.4	77.4 ± 9.3	0.772
Hypertension status ^a,e^			0.810			0.339			1.0
Hypertensive	24.5%	11.3%		20.6%	33.3%		12.2%	8.3%	

^a^—results are expressed as mean ± sd; ^b^—results are presented as frequency (%); ^c^—Independent *t*-test; ^d^—Pearson’s χ^2^; ^e^—Independent-Samples Mann-Whitney U Test.

**Table 2:** Urinary excretion and nutrient/food intake of participants according to sex and adherence to Mediterranean Diet.

				Adherence to Mediterranean Diet					
				Male			Female		
	Males *n* = 49	Females *n* = 53	*p*	Low-moderate *n* = 34	High *n* = 15	*p*	Low-moderate *n* = 41	High *n* = 12	*p*
Urinary excretion									
Urinary Sodium excretion (mg/day) ^a,c,f^	3850 ± 1317	2630.7 ± 994	<0.001	3868 ± 1390	3807 ± 1179	0.883	2591 ± 966	2768 ± 1116	0.592
Urinary Potassium excretion (mg/day) ^a,c,f^	2932 ± 794	2382 ± 614	<0.001	2930 ± 849	2938 ± 681	0.973	2373 ± 660	2412 ± 443	0.852
Urinary sodium-to-potassium ratio (mmol/mmoL) ^a,c^	2.38 ± 1	1.95 ± 1	0.024	2.40 ± 1	2.33 ± 1	0.841	1.94 ± 1	1.96 ± 1	0.943
Creatinine (mg/kg/day) ^a,c^	22 ± 4	18.3 ± 3	<0.001	22 ± 4	21 ± 2.	0.232	18 ± 3	18 ± 3	0.751
Nutrient and dietary intake									
Sodium intake (mg/24 h) ^a,c^	4230 ± 1447	2891 ± 1092	<0.001	4251 ± 1528	4184 ± 1295	0.883	2847 ± 1062	3041 ± 1227	0.592
Potassium intake (mg/24 h) ^a,c^	3808 ± 1031	3093 ± 798	<0.001	3805 ± 1102	3816 ± 885	0.973	3082 ± 857	3132 ± 576	0.852
Sodium-to-potassium ratio (mmoL/mmoL) ^a,c^	2.02 ± 0.93	1.65 ± 0.67	0.024	2.03 ± 0.94	2.0 ± 0.95	0.841	1.64 ± 0.70	1.66 ± 0.61	0.943
Salt intake (g/day) ^a,c^	10.8 ± 3.7	7.4 ± 2.78	<0.001	10.8 ± 3.88	10.64 ± 3.29	0.781	7.2 ± 2.7	7.7 ± 3.1	0.705
Sodium intake >2000 mg ^b,d^	94%	64%	<0.001	91.2%	100%	0.543	65.9%	58.3%	0.736
Potassium intake <3510 mg ^b,d^	41%	70%	0.003	41.2%	40%	1.0	68.3%	75%	0.737
Energy (kcal/day) ^a,c^	2131 ± 680.0	2113 ± 728.1	0.902	1942 ± 540.4	2559 ± 784.3	0.002	1948 ± 589.3	2678 ± 890.6	0.002
Vegetables (g/day) ^a,e^	228 ± 133	217 ± 201	1.000	202 ± 104	262 ± 164	0.44	199 ± 104	315 ± 303	<0.001
Legumes (g/day) ^a,e^	37 ± 54	28 ± 45	0.428	26 ± 51	78 ± 55	0.028	26 ± 41	73 ± 49	0.034
Fruits (g/day) ^a,e^	216 ± 165	272 ± 203	0.047	204 ± 109	284 ± 226	0.013	255 ± 152	426 ± 287	0.014
Nuts (g/day) ^a,e^	5 ± 57	10 ± 47	0.771	4.7 ± 20	30 ± 91	0.004	4.7 ± 23	30 ± 85	0.003
Whole grain (g/day) ^a,e^	33 ± 43	38 ± 42	1.000	21 ± 36	55 ± 47	0.002	21 ± 40	50 ± 45	0.105
Fish (g/day) ^a,e^	74 ± 48	84 ± 64	0.837	64 ± 35	90 ± 64	0.068	81 ± 41	113 ± 103	0.026
Ratio MUFA/SFA ^a,e^	1.6 ± 0.5	1.7 ± 0.5	1.000	1.48 ± 0.35	2.12 ± 0.58	<0.001	1.6 ± 0.47	2.13 ± 0.46	0.074
Red meat (g/day) ^a,e^	56 ± 29	51 ± 33	0.332	58 ± 28	54 ± 31	0.625	53 ± 29	31 ± 45	0.413
Alcohol (mg/day) ^a,e^	6.7 ± 11.8	2.5 ± 4.6	0.006	6.7 ± 13	5.8 ± 7	0.630	5.0 ± 5	1.6 ± 2	0.068

^a^—results are expressed as mean ± sd; ^b^—results are presented as frequency (%); ^c^—Independent *t*-test; ^d^—Pearson’s χ^2^; ^e^—Independent-Samples Mann-Whitney U Test; ^f^—To convert to mmol/24 h divide sodium (mg) and potassium (mg) respectively for 23 or 39 (mmol = mg/atomic weight).

**Table 3:** Male odds ratios for the association between high adherence to MD and sodium, potassium, and sodium-to-potassium ratio last tertile.

	Crude Model			Model 1 ^1^			Model 2 ^2^			Model 3 ^3^		
	OR	95% CI	*p*	OR	95% CI	*p*	OR	95% CI	*p*	OR	95% CI	*p*
Sodium excretion tertile												
1st Tertile	1	(Reference)			(Reference)			(Reference)			(Reference)	
2nd Tertile	2.625	0.574–11.998	0.213	2.681	0.578–12.438	0.208	2.146	0.383–12.021	0.385	2.539	0.345–18.706	0.360
3th Tertile	0.857	0.176–4.186	0.849	0.728	0.140–3.781	0.705	0.485	0.071–3.317	0.461	0.364	0.045–2.937	0.343
Potassium excretion tertile												
1st Tertile	1	(Reference)			(Reference)			(Reference)			(Reference)	
2nd Tertile	2.625	0.574–11.998	0.213	2.200	0.435–11.126	0.341	2.746	0.416–18.115	0.294	5.085	0.517–50.011	0.163
3th Tertile	0.857	0.176–4.186	0.849	0.654	0.113–3.783	0.636	0.792	0.111–5.630	0.815	0.839	0.102–6.883	0.870
Sodium-to-potassium ratio excretion tertile												
1st Tertile	1	(Reference)			(Reference)			(Reference)			(Reference)	
2nd Tertile	2.667	0.608–11.703	0.194	2.744	0.606–12.437	0.190	3.980	0.673–23.526	0.128	3.068	0.499–18.855	0.226
3th Tertile	0.692	0.128–3.752	0.670	0.672	0.121–3.719	0.649	0.639	0.085–4.783	0.663	0.503	0.057–4.420	0.536

^1^ Model 1: adjusted for age and Body Mass Index; ^2^ Model 2: model 1, and additionally adjusted for energy intake; ^3^ Model 3: model 2, and additionally adjusted for hypertension, physical activity, and education. OR = Odds Ratio.

**Table 4:** Female Odds Ratios for the association between high adherence to MD and sodium, potassium, and sodium-to-potassium ratio last tertile.

Crude Model				Model 1 ^1^			Model 2 ^2^			Model 3 ^3^		
	OR	95% CI	*p*	OR	95% CI	*p*	OR	95% CI	*p*	OR	95% CI	*p*
Sodium excretion tertile												
1st Tertile	1	(Reference)			(Reference)			(Reference)			(Reference)	
2nd Tertile	0.300	0.049–1.820	0.190	0.282	0.045–1.759	0.175	0.288	0.041–2.010	0.209	0.122	0.012–1.267	0.078
3th Tertile	0.923	0.213–4.003	0.915	0.821	0.154–4.384	0.817	1.310	0.195–8.810	0.781	4.135	0.302–56.555	0.288
Potassium excretion tertile												
1st Tertile	1	(Reference)			(Reference)			(Reference)			(Reference)	
2nd Tertile	2.727	0.557–13.365	0.216	2.381	0.462–12.287	0.300	4.767	0.550–41.276	0.156	5.272	0.435–63.932	0.192
3th Tertile	1.000	0.173–5.772	1.000	0.987	0.170–5.740	0.988	1.388	0.176–10.914	0.756	1.749	0.178–17.163	0.631
Sodium-to-potassium ratio excretion tertile												
1st Tertile	1	(Reference)			(Reference)			(Reference)			(Reference)	
2nd Tertile	1.795	0.356–9.054	0.479	1.685	0.314–9.044	0.543	1.396	0.238–8.182	0.711	1.099	0.155–7.791	0.925
3th Tertile	1.333	0.251–7.084	0.736	0.915	0.132–6.344	0.928	0.945	0.120–7.462	0.957	1.800	0.178–18.173	0.618

^1^ Model 1: adjusted for age and Body Mass Index; ^2^ Model 2: model 1, and additionally adjusted for energy intake; ^3^ Model 3: model 2, and additionally adjusted for hypertension, physical activity, and education. OR = Odds Ratio.

## Data Availability

The authors can share data upon request.
